# Rapid CD8^+^ Function Is Critical for Protection of Neonatal Mice from an Extracellular Bacterial Enteropathogen

**DOI:** 10.3389/fped.2016.00141

**Published:** 2017-01-09

**Authors:** David T. Siefker, Becky Adkins

**Affiliations:** ^1^Department of Pediatrics, Le Bonheur Children’s Medical Center, Memphis, TN, USA; ^2^Department of Microbiology and Immunology, University of Miami Miller School of Medicine, Miami, FL, USA

**Keywords:** neonatal, enteropathogens, CD8^+^ T cells, immunity, innate, IFN-gamma

## Abstract

Both human and murine neonates are characteristically highly susceptible to bacterial infections. However, we recently discovered that neonatal mice are surprisingly highly resistant to oral infection with *Yersinia enterocolitica*. This resistance was linked with activation of both innate and adaptive responses, involving innate phagocytes, CD4^+^ cells, and B cells. We have now extended these studies and found that CD8^+^ cells also contribute importantly to neonatal protection from *Y. enterocolitica*. Strikingly, neonatal CD8^+^ cells in the mesenteric lymph nodes (MLN) are rapidly mobilized, increasing in proportion, number, and IFNγ production as early as 48 h post infection. This early activation appears to be critical for protection since B2m^−/−^ neonates are significantly more susceptible than wt neonates to primary *Y. enterocolitica* infection. In the absence of CD8^+^ cells, *Y. enterocolitica* rapidly disseminated to peripheral tissues. Within 48 h of infection, both the spleens and livers of B2m^−/−^, but not wt, neonates became heavily colonized, likely leading to their deaths from sepsis. In contrast to primary infection, CD8^+^ cells were dispensable for the generation of immunological memory protective against secondary infection. These results indicate that CD8^+^ cells in the neonatal MLN contribute importantly to protection against an extracellular bacterial enteropathogen but, notably, they appear to act during the early innate phase of the immune response.

## Introduction

Neonates and infants are commonly highly susceptible to infectious diseases. Many of these diseases are caused by bacterial pathogens, which are largely naturally acquired by oral ingestion. Because of the many similarities in immunity in early life between mice and humans (1, 2), neonatal mice provide a reasonably faithful and experimentally convenient model system for studying infection with bacterial enteropathogens. Indeed, as in humans, mouse neonates are very sensitive to oral infection with a number of bacterial enteropathogens, including *Salmonella typhimurium* (3, 4), *Helicobacter pylori* (5–7), *Shigella flexneri* (8–10), *Vibrio cholera* (11, 12), and the Enteropathogenic *E. coli*-related *Citrobacter rodentium* (13–16). Often, these susceptibilities are linked to quantitative or qualitative differences in neonatal and adult responses involving both the innate and adaptive gastrointestinal immune systems.

In contrast to all other descriptions of infections in neonatal mice, our laboratory found that 7-day-old murine neonates are highly resistant to orogastric infection with the extracellular enteropathogen *Yersinia enterocolitica* (17). *Y. enterocolitica* disseminates to the mesenteric lymph nodes (MLN), and analyses of immune function in that site revealed robust responses involving both the innate and adaptive arms of immunity (18, 19). Proinflammatory cytokine gene expression was highly induced, and innate phagocytes infiltrated the MLN in high numbers. Mature or supra-mature adaptive responses were also detected. We demonstrated that CD4 cell Th1 and Th17 function were both critical for protection of neonates and serum antibody responses of similar magnitude and avidity to those in adults were observed. Finally, and strikingly, neonates developed protective immunity against subsequent exposure as adults to a lethal dose of the bacterium.

These results indicated that protective responses against *Y. enterocolitica* in early life involve multiple innate and adaptive cell types. In these initial experiments, we largely ignored CD8 cells since these cells are more commonly associated with viral or intracellular bacterial infections. However, in the course of these studies, we noted that a large proportion of CD8 cells in the MLN of uninfected neonates expressed the proliferative antigen Ki67. Upon infection, CD8^+^, but not CD4^+^ cells, increased rapidly in proportion and IFNγ production. B2m^−/−^ neonates were more susceptible to infection, compared with wt neonates, indicating that CD8^+^ cells were required for survival of primary infection. The susceptibility of B2m^−/−^ neonates was linked to the early dissemination of the bacteria to peripheral organs. Last, although required for primary infection, CD8^+^ cells were dispensable for survival of secondary infection. These results indicate that neonatal CD8^+^ cells may play an important early, innate-like role in survival to primary infection with *Y. enterocolitica* but they are not necessary for the development of protective memory in neonates.

## Materials and Methods

### Mice

Adult C57BL/6 and B2m-deficient mice (B6.129P2-B2m^tm1-Unc^/J) were purchased from Jackson Laboratories. All mice were bred and housed under barrier conditions in the Division of Veterinary Resources of the University of Miami Miller School of Medicine. Mice were regularly screened for specific common pathogens. Adult mice (6–10 weeks of age) and neonatal mice (7 days of age) were used in experiments. All mice were handled in compliance with the Institutional Animal Care and Use Committee (IACUC) of the University of Miami Miller School of Medicine, Miami, FL, USA.

### Bacterial Infections

Wild-type high-virulence *Y. enterocolitica* A127/90 serotype 0:8 biotype IB was originally provided by G. R. Cornelis (Universität Basel, Basel, Switzerland). For infection, bacterial frozen stocks (17) were washed twice with Hank’s Balanced Salt Solution (HBSS, Gibco, Grand Island, NY, USA), diluted to the desired concentration, and inoculated with the indicated doses. Adults were inoculated orogastrically with a 22-gauge, round-tipped feeding needle (Fine Science Tools, Foster City, CA, USA) attached to a 1-ml syringe (Becton Dickinson, Franklin Lakes, NJ, USA). Neonates were inoculated orogastrically with PE-10 tubing (polyethylene tubing with an outside diameter of 0.61 mm) (Clay Adams, Sparks, MD, USA) attached to a 30-gauge needle and Hamilton syringe (20). The actual administered dose was determined by plating serial dilutions of the suspensions on Luria Broth plates and incubating for 48 h at 27°C.

### Cell Staining, Antibodies, and Flow Cytometry Analysis

Individual MLN from neonates and adults were harvested and placed in cold HBSS containing 1% calf serum (Gibco), 10 mM HEPES (Gibco), and 4 mM sodium azide. Cell suspensions were prepared by mincing tissues with scissors and pressing cells through wire mesh with 74 µm pore size (Compass Wire, Westville, NJ, USA). Cells were incubated in mouse Fc Block (CD16/CD32; BD Pharmingen, San Diego, CA, USA) for 5 min on ice, followed by a 30-min incubation with fluorochrome-conjugated antibodies specific for CD4, CD8, Ki67, or TCRαβ (BD Pharmingen). For intracellular cytokine staining, cells were activated with 50 ng/ml PMA and 0.5 µM ionomycin in the presence of 5 µg/ml brefeldin A, fixed and permeabilized, and stained with fluorochrome-conjugated anti-IFNγ (BD Pharmingen). Samples were run on a Becton Dickinson LSR II flow cytometer and analyzed with FlowJo flow cytometry analysis software.

### Bacterial Enumeration from Organs of Infected Mice

To measure *Y. enterocolitica* titers, tissues were weighed and homogenized in HBSS using a Seward Biomaster 80 Stomacher (Brinkman, Westbury, NY, USA) for 4 min at high speed. For small intestine mucosa-associated *Y. enterocolitica* titers, the small intestine contents were flushed with HBSS prior to homogenization. Individual MLN were homogenized in 400 μl (neonates) or 500 μl (adults) of HBSS using a VWR disposable pellet mixer with cordless motor (VWR International). *Y. enterocolitica* titers were enumerated by plating dilutions of homogenates on Difco Yersinia Selective Agar Base plates (Becton Dickinson, Sparks, MD, USA).

### Statistical Analyses

All experiments were performed at least two times. Statistical tests were performed using GraphPad Prism software, as follows: unpaired *t* test for the Ki67/CD4/CD8/IFNγ staining experiments; Mann–Whitney test for the bacterial colonization experiments; Log-rank (Mantel–Cox) test for the survival experiments. The significant threshold was *P* ≤ 0.05.

## Results

### Rapid Responses of Neonatal MLN CD8^+^ Cells following Oral *Y. enterocolitica* Infection

**Figure 1:**
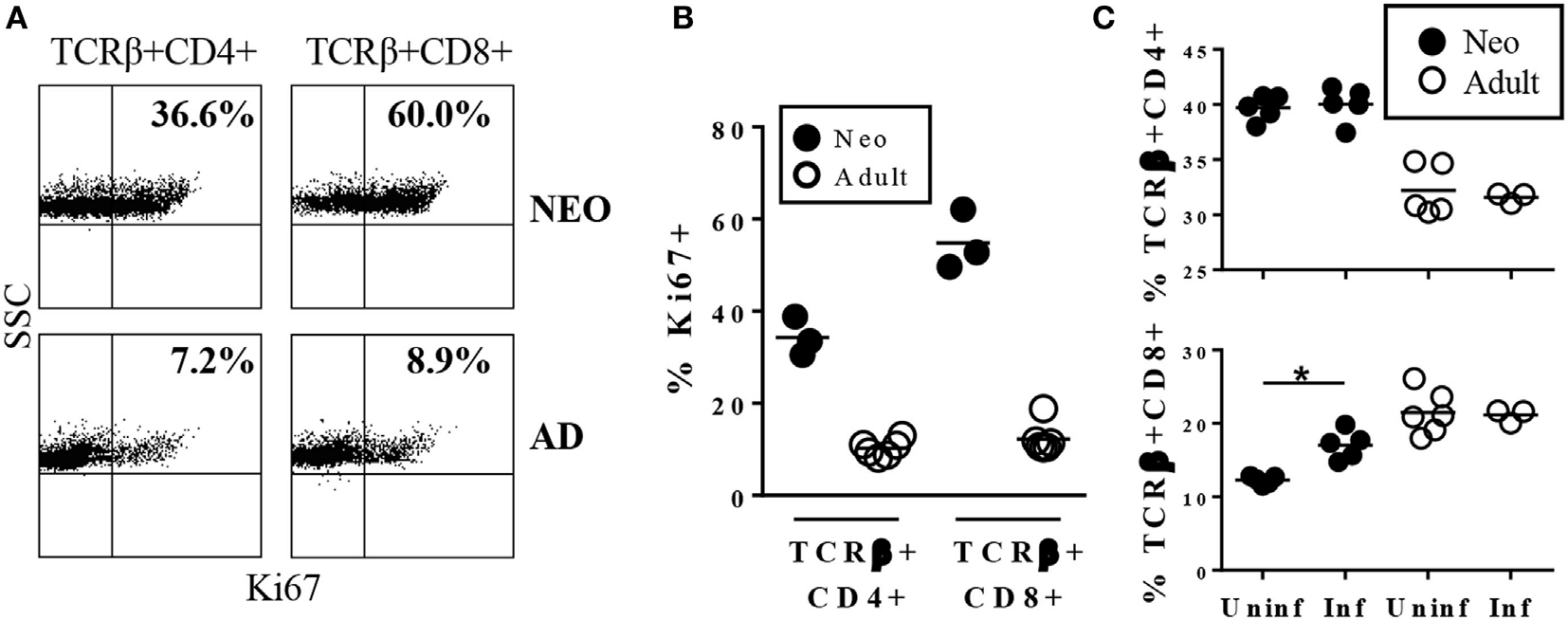
**Neonatal TCRβ^+^CD8^+^ cells in the mesenteric lymph nodes (MLN) are cycling in uninfected animals and rapidly increase in response to infection with *Yersinia enterocolitica***. **(A)** Representative profile of MLN cells from uninfected 7-day-old or adult mice stained for TCRβ, CD4, CD8, and Ki67. **(B)** The percentages of Ki67^+^ among TCRβ^+^CD4^+^ or TCRβ^+^CD8^+^ cells in uninfected individual neonatal or adult MLN. **(C)** The percentages of TCRβ^+^CD4^+^ or TCRβ^+^CD8^+^ MLN cells in individual mice 1 day post infection with 5 × 10^8^ CFU *Y. enterocolitica*.

In the course of characterizing immune responses to *Y. enterocolitica* infection, we compared MLN cells from uninfected neonates and adults for expression of the proliferation antigen Ki67. Approximately 10% of both CD4^+^ and CD8^+^ cells in adult MLN expressed the Ki67 antigen. A much greater proportion of both T cell types were Ki67^+^ in neonatal MLN (Figures 1A,B). This is consistent with previous studies in which neonatal T cells in mouse spleen and cord blood were found to be spontaneously proliferating at a higher rate than in adults (21, 22). However, the frequency of proliferating cells in the MLN, especially among the CD8^+^ population, is markedly higher than in blood or spleen.

The substantial endogenous proliferation of CD8^+^ cells in uninfected neonates indicated that this population may pre-exist in a state poised for rapid responsiveness to invasive intestinal pathogens. Indeed, within 24 h of infection with *Y. enterocolitica*, there was a significant increase in the proportion of neonatal CD8^+^ cells while percentages of neonatal CD4^+^ and adult CD4^+^ and CD8^+^ did not increase (Figure 1C). The absolute numbers showed a similar pattern, although neonatal CD4^+^ cells also increased by fivefold while CD8^+^ cell numbers increased eightfold. Neither population increased in absolute numbers in adults.

**Figure 2:**
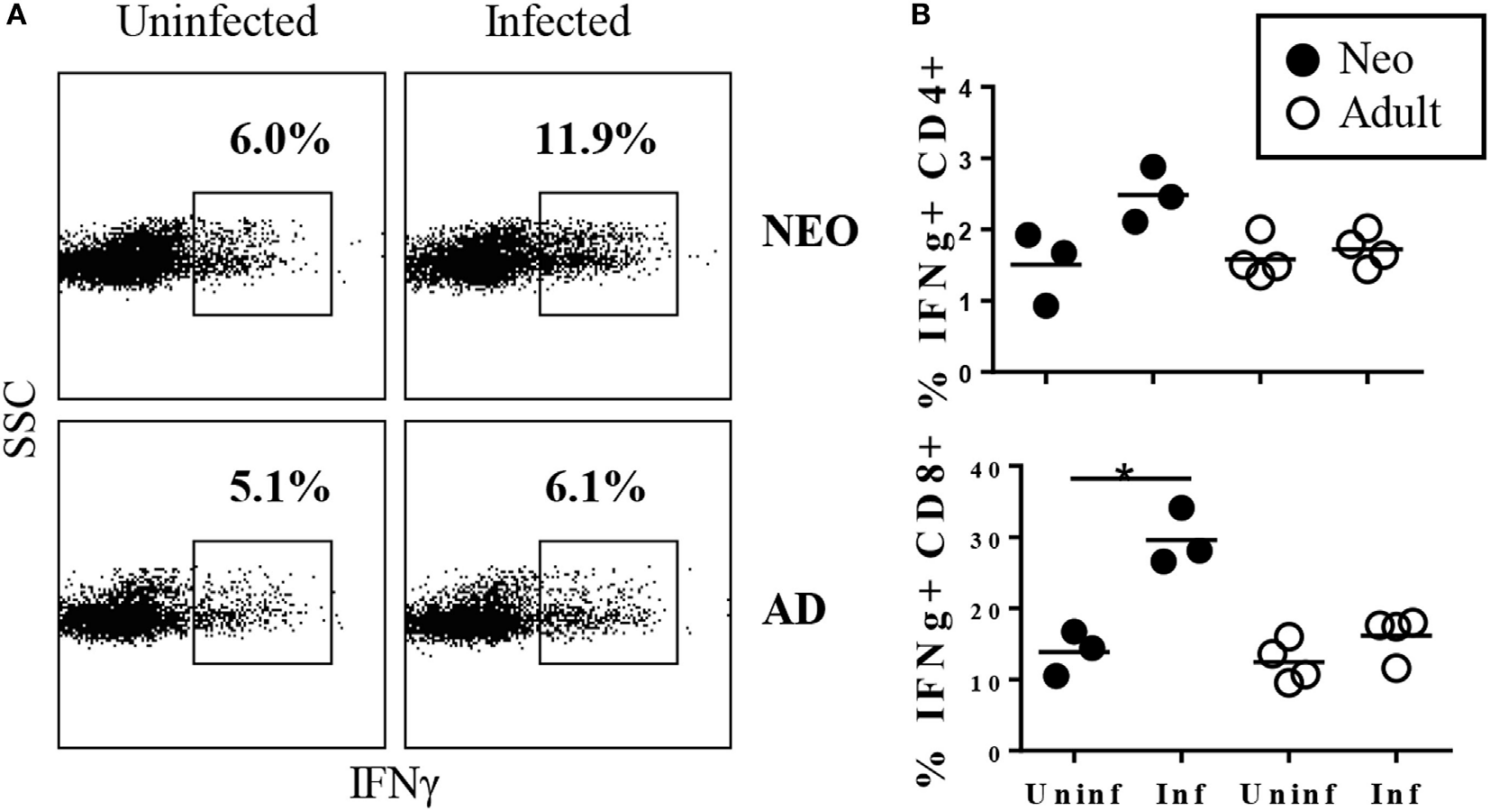
**Rapid increase in the percentages of TCRβ^+^CD8^+^ cells producing IFNγ in neonatal mesenteric lymph nodes (MLN) after infection with *Yersinia enterocolitica***. Neonatal and adult mice were infected with 5 × 10^8^ CFU *Y. enterocolitica*. Forty-eight hours later, MLN cells were prepared, stimulated with PMA + ionomycin, in the presence of brefeldin A, for 4 h, and then subjected to intracellular cytokine staining. **(A)** Frequencies of IFNγ^+^ cells among total lymph node cells in uninfected and infected neonatal and adult animals. Gates for IFNγ were determined by comparison with isotype-matched control stains. **(B)** The frequencies of IFNγ-producing cells within the TCRβ^+^CD4^+^ or TCRβ^+^CD8^+^ populations. Each point for neonates represents a pool of 2–4 animals; a total of eight neonates were examined. Each point for adults represents an individual animal. Differences between uninfected and infected animals are non-significant unless indicated with *.

These results indicated that the neonatal CD8^+^ population was able to expand rapidly in response to *Y. enterocolitica* infection. The next question was whether this expansion was accompanied by effector function. A prominent effector function of CD8^+^ cells is the production of IFNγ. Our previous studies had shown that IFNγ is required for survival of neonates to *Y. enterocolitica* infection (18) and that IFNγ production by CD4^+^ cells was an important component of protection. However, IFNγ-producing CD4^+^ cells most likely act in the later stages of primary infection, during the adaptive phase of the response, since death in CD4-deficient neonates is delayed until ≥15 days post infection. In striking contrast, IFNγ-deficient neonates die rapidly, within 7 days of infection. Together, these results suggest that IFNγ may provide important protection both early and late post infection. Thus, we proposed that early activated CD8^+^ cells may be a rapid source of IFNγ production. We first examined early IFNγ production among total MLN cells and found an increase in total cytokine-producing cells in neonates, but not adults (Figure 2A). Indeed, a significant increase in IFNγ-production was selectively observed in the neonatal CD8^+^ population (Figure 2B). Thus, *Y. enterocolitica* infection of neonates is characterized by the rapid mobilization of CD8^+^ MLN populations with IFNγ-producing effector function.

### Neonatal CD8^+^ Cells Are Essential for Survival to Primary Infection and for Preventing Early Systemic Dissemination of *Y. enterocolitica*

**Figure 3:**
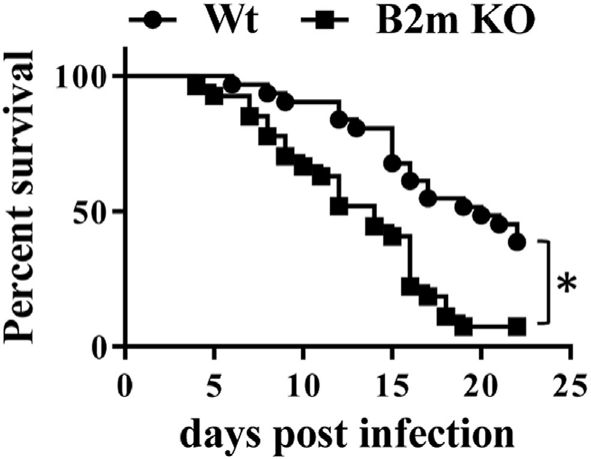
**CD8^+^ cells are critical for survival of neonatal mice to primary *Yersinia enterocolitica* infection**. The 7-day-old wt or B2m^−/−^ pups were orally infected with 1 × 10^7^ CFU *Y. enterocolitica* and survival was monitored. A total of 33 wt and 27 B2m^−/−^ pups was analyzed in ≥3 separate experiments. *P* = 0.0004 in the Log-rank (Mantel–Cox) test.

Our results thus far indicated that neonates infected with *Y. enterocolitca* mount early CD8^+^ responses. To test whether CD8^+^ cells were essential for protection against infection, survival of wt and B2m^−/−^ neonates to a threshold lethal dose of *Y. enterocolitica* was compared. As shown in Figure 3, B2m^−/−^ neonates were more susceptible to lethal infection and, notably, most deaths occurred prior to 14 days post infection—i.e., well before the time that CD4^−/−^ neonates begin to succumb (18). In these experiments, we used female and male neonates indiscriminately but subset analyses showed that there were no differences in survival of male or female neonates in either wt or B2m^−/−^ neonates. Therefore, CD8^+^ cells appear to play an important role in the survival of neonates, regardless of gender.

**Figure 4:**
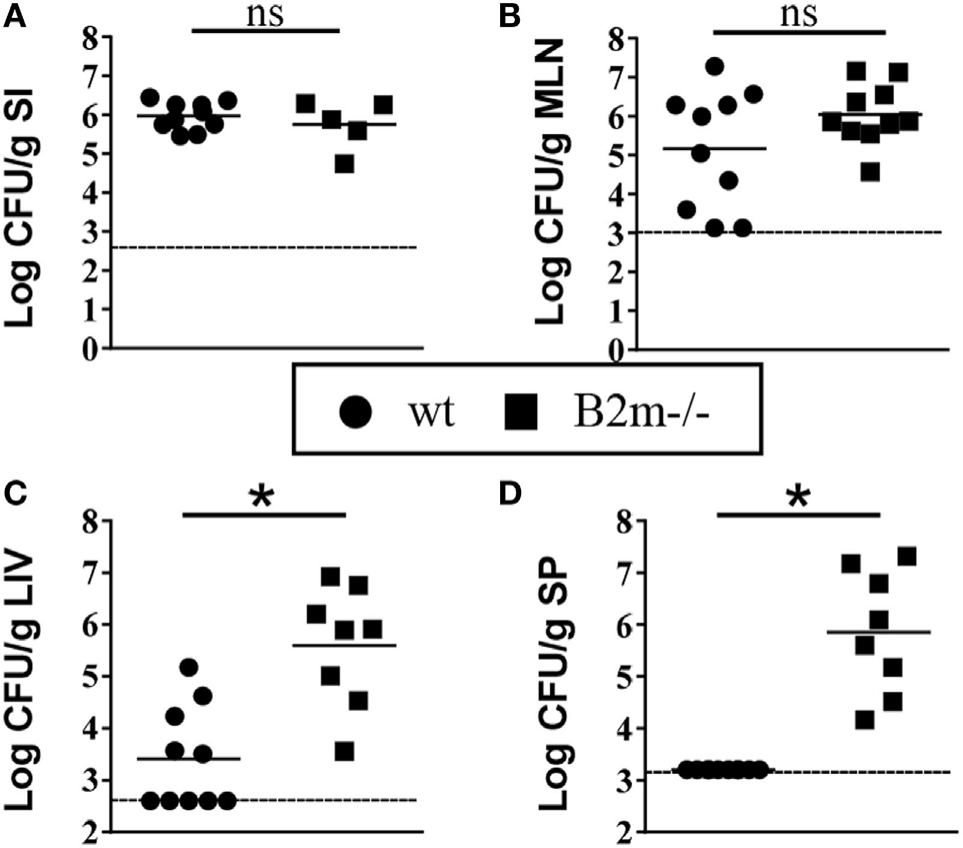
**Enhanced early dissemination to peripheral tissues in B2m^−/−^ neonates**. 7-day-old wt and B2m^−/−^ pups were orally infected with 5 × 10^8^ CFU *Yersinia enterocolitica*, and CFU in **(A)** small intestines (minus contents) (SI), **(B)** mesenteric lymph nodes (MLN), **(C)** liver (LIV), and **(D)** spleen (SP) were measured 48 h post infection. Each point represents an individual mouse; the dashed lines indicate the limits of detection in each assay.

*Yersinia enterocolitica* replicates in the small intestines and routinely invades to the MLN but only systemically disseminates if the dose of infection is very high in wt animals or if animals are immunocompromised (23). Significant systemic dissemination most likely leads to death *via* septic shock. To investigate the potential importance of CD8^+^ cells in confining *Y. enterocolitica* to the intestinal tissues, we compared early colonization of intestinal and systemic organs in infected wt and B2m^−/−^ neonates. 48 h post infection, colonization levels of the small intestines and MLN were similar in wt and B2m^−/−^ neonates (Figures 4A,B). However, at this relatively early time point, both the liver and spleen showed massive bacterial infiltration only in the B2m^−/−^ neonates (Figures 4C,D). These results indicate that the early activation of CD8^+^ cells in the neonatal MLN may be critical for preventing the systemic spread of *Y. enterocolitica*.

### Protective Memory Responses Are Generated in Neonates in the Absence of CD8^+^ Cells

**Figure 5:**
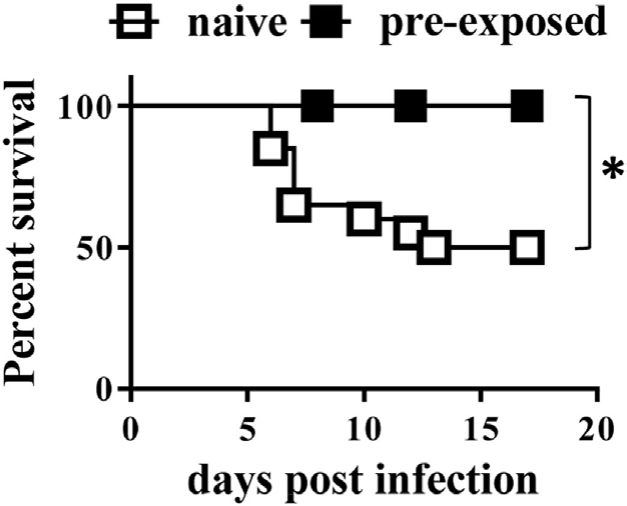
**Neonatal mice develop protective memory responses in the absence of CD8^+^ cells**. 7-day-old B2m^−/−^ mice were infected with 1 × 10^4^ CFU *Yersinia enterocolitica*. Eight weeks later, the mice were challenged with 1 × 10^5^ CFU *Y. enterocolitica*; in parallel, naïve age-matched B2m^−/−^ controls were similarly challenged. A total of 6 pre-infected and 20 naïve mice were analyzed in two separate experiments. *P* = 0.0239, using the Log-rank (Mantel–Cox) test.

In adult mice, CD8^+^ cells appear to be essential for protection against secondary infection with the closely related bacterium *Yersinia pseudotuberculosis* (24). To test whether this was also the case in neonates, B2m^−/−^ neonates were infected with a sublethal dose (1 × 10^4^ CFU) of *Y. enterocolitica*. Uninfected littermates were kept as control mice. Eight weeks later, all mice were challenged with 1 × 10^5^ CFU of *Y. enterocolitica*, and survival was monitored. At this challenge dose, approximately 50% of the control, previously uninfected, mice survived (Figure 5). However, 100% of the B2m^−/−^ mice previously infected as neonates survived the infection. Therefore, CD8^+^ cells are not required for either the development of immunological memory in neonates or for its manifestation in response to secondary challenge.

## Discussion

The results presented here demonstrate that CD8^+^ cells are critical for protection of neonates against oral exposure to an extracellular bacterial enteropathogen. The protective effects appear to manifest early after infection since CD8^+^ cells in the MLN selectively increase within 24 h of infection and over ¼ of the cells are producing IFNγ just 48 h post infection. Moreover, CD8^+^ cells appear to be important for the early containment of bacteria within the intestines; the spleens and livers of B2m^−/−^ neonates were heavily colonized by bacteria as early as 48 h post infection. Therefore, unlike in their typical adaptive role, neonatal CD8^+^ cells in intestinal lymphoid tissues act during the early, innate phase of the response, providing an important rapid antibacterial function.

CD8^+^ cell function in neonates has been mostly studied in response to infection with viruses, including influenza virus (25, 26), herpes simplex virus (27–29), respiratory syncytial virus (30), cytomegalovirus (31, 32), lymphocytic choriomeningitis virus (33), adenovirus (34), and Cas-Br-E murine leukemia virus (35–37). Most often, these viruses have been introduced either systemically or through a pulmonary route. Common, although not exclusive, observations are that the CD8 cell response is delayed relative to that of adults, lytic and/or IFNγ-secreting activities are diminished, the repertoire appears to be limited, and neonatal CD8^+^ memory responses are poor. In all cases, however, neonatal CD8^+^ cell activity was measured during the adaptive phase of the response. There are far fewer studies on neonatal CD8 cell function in response to bacterial infection and those are limited to systemic infection of neonates with the intracellular pathogen *Listeria monocytogenes* (38, 39). Thus, we believe that the neonatal response to *Y. enterocolitica* represents the first description of neonatal CD8^+^ cells acting in a protective manner during the innate phase of the response against an extracellular bacterial enteropathogen.

Although CD8 cells had not previously been implicated in enterobacterial infection in neonates, a role for these cells in adult *Yersinia* infection has been described (24). Like our observations in neonates, it was reported that oral infection with *Y. pseudotuberculosis* led to increased colonization of peripheral tissues in B2m^−/−^ adults. However, we found rapid (≤48 h) increased colonization in neonatal B2m^−/−^ mice whereas the increase in adults was not detected until 14 days post inoculation, during the adaptive phase of immunity. In addition, we found that CD8 cells were not required for protective memory responses whereas in adults, anti-CD8 treatment greatly compromised memory responses. Overall, while CD8 cells may contribute to immunity against *Yersinia* infection in both neonates and adults, the roles these cells play and when they act differ in early life and adulthood.

The mechanisms underlying the early responses of neonatal CD8 cells in *Yersinia enterocolitica* infection are not yet fully understood. One finding that may provide some insight is the observation that >50% of neonatal MLN CD8^+^ cells express proliferating antigens in uninfected, resting animals. Due to the lymphopenic state of neonates, homeostatic proliferation of both CD4^+^ and CD8^+^ cells has been previously described in neonatal spleen (21) and in human cord blood (22). In both cases, as we see here, a greater proportion of CD8^+^ cells are cycling relative to CD4^+^ cells. However, the percentages of CD8^+^ cells in cycle in the MLN is approximately fivefold higher than that in the blood or spleen, perhaps due to the proximity of ongoing colonization by the commensal microbiota (40). Proliferation in lymphopenic hosts leads to the capacity for rapid induction of IFNγ expression (41, 42)—in our case, the IFNγ appears to be elicited upon exposure to bacterial enteropathogen. In that regard, human neonatal CD8^+^ cells have been shown to rapidly respond to TLR2 or TLR5 stimulation with increased proliferation and cytokine production (43), and it is possible that murine neonatal CD8^+^ cells in the MLN are similarly responding to PAMPs expressed by *Y. enterocolitica*. Thus, neonatal CD8 cells may perform both adaptive functions, in response to virus or intracellular bacteria, and innate functions when exposed to extracellular pathogens, especially at mucosal sites.

## Author Contributions

DS performed many of the experiments in partial fulfillment of his doctoral degree. He contributed significantly to the intellectual development of the overall project and the manuscript. BA, the principal investigator, oversaw the scholarly and technical progress of the project and wrote the manuscript.

## Conflict of Interest Statement

The authors declare that the research was conducted in the absence of any commercial or financial relationships that could be construed as a potential conflict of interest.

## References

[1] Dowling DJ Levy O. Ontogeny of early life immunity. Trends Immunol (2014) 35:299–310. 10.1016/j.it.2014.04.007 24880460 PMC4109609

[2] Prabhudas M Adkins B Gans H King C Levy O Ramilo O Challenges in infant immunity: implications for responses to infection and vaccines. Nat Immunol (2011) 12:189–94. 10.1038/ni0311-189 21321588

[3] Zhang K Dupont A Torow N Gohde F Leschner S Lienenklaus S Age-dependent enterocyte invasion and microcolony formation by *Salmonella*. PLoS Pathog (2014) 10:e1004385. 10.1371/journal.ppat.1004385 25210785 PMC4161480

[4] Rhee SJ Walker WA Cherayil BJ. Developmentally regulated intestinal expression of IFN-gamma and its target genes and the age-specific response to enteric *Salmonella* infection. J Immunol (2005) 175:1127–36. 10.4049/jimmunol.175.2.1127 16002714

[5] Oertli M Sundquist M Hitzler I Engler DB Arnold IC Reuter S DC-derived IL-18 drives Treg differentiation, murine *Helicobacter pylori*-specific immune tolerance, and asthma protection. J Clin Invest (2012) 122:1082–96. 10.1172/JCI61029 22307326 PMC3287234

[6] Arnold IC Dehzad N Reuter S Martin H Becher B Taube C *Helicobacter pylori* infection prevents allergic asthma in mouse models through the induction of regulatory T cells. J Clin Invest (2011) 121:3088–93. 10.1172/JCI45041 21737881 PMC3148731

[7] Arnold IC Lee JY Amieva MR Roers A Flavell RA Sparwasser T Tolerance rather than immunity protects from *Helicobacter pylori*-induced gastric preneoplasia. Gastroenterology (2011) 140:199–209. 10.1053/j.gastro.2010.06.047 20600031 PMC3380634

[8] Fernandez MI Thuizat A Pedron T Neutra M Phalipon A Sansonetti PJ. A newborn mouse model for the study of intestinal pathogenesis of shigellosis. Cell Microbiol (2003) 5:481–91. 10.1046/j.1462-5822.2003.00295.x 12814438

[9] Fernandez MI Regnault B Mulet C Tanguy M Jay P Sansonetti PJ Maturation of paneth cells induces the refractory state of newborn mice to *Shigella* infection. J Immunol (2008) 180:4924–30. 10.4049/jimmunol.180.7.4924 18354217

[10] Shim DH Ryu S Kweon MN. Defensins play a crucial role in protecting mice against oral *Shigella flexneri* infection. Biochem Biophys Res Commun (2010) 401:554–60. 10.1016/j.bbrc.2010.09.100 20888321

[11] Bishop AL Patimalla B Camilli A. *Vibrio cholerae*-induced inflammation in the neonatal mouse cholera model. Infect Immun (2014) 82:2434–47. 10.1128/IAI.00054-14 24686062 PMC4019157

[12] Klose KE. The suckling mouse model of cholera. Trends Microbiol (2000) 8:189–91. 10.1016/S0966-842X(00)01721-2 10754579

[13] D’Arienzo R Maurano F Mazzarella G Luongo D Stefanile R Ricca E *Bacillus subtilis* spores reduce susceptibility to *Citrobacter rodentium*-mediated enteropathy in a mouse model. Res Microbiol (2006) 157:891–7. 10.1016/j.resmic.2006.06.001 17005378

[14] Barthold SW Coleman GL Jacoby RO Livestone EM Jonas AM. Transmissible murine colonic hyperplasia. Vet Pathol (1978) 15:223–36. 10.1177/030098587801500209 664189

[15] Gareau MG Wine E Reardon C Sherman PM. Probiotics prevent death caused by *Citrobacter rodentium* infection in neonatal mice. J Infect Dis (2010) 201:81–91. 10.1086/648614 19961304

[16] Rodrigues DM Sousa AJ Johnson-Henry KC Sherman PM Gareau MG. Probiotics are effective for the prevention and treatment of *Citrobacter rodentium*-induced colitis in mice. J Infect Dis (2012) 206:99–109. 10.1093/infdis/jis177 22430833

[17] Echeverry A Schesser K Adkins B. Murine neonates are highly resistant to *Yersinia enterocolitica* following orogastric exposure. Infect Immun (2007) 75:2234–43. 10.1128/IAI.01681-06 17325052 PMC1865740

[18] Echeverry A Saijo S Schesser K Adkins B. *Yersinia enterocolitica* promotes robust mucosal inflammatory T-cell immunity in murine neonates. Infect Immun (2010) 78:3595–608. 10.1128/IAI.01272-09 20515925 PMC2916279

[19] Siefker DT Echeverry A Brambilla R Fukata M Schesser K Adkins B. Murine neonates infected with *Yersinia enterocolitica* develop rapid and robust proinflammatory responses in intestinal lymphoid tissues. Infect Immun (2014) 82:762–72. 10.1128/IAI.01489-13 24478090 PMC3911368

[20] Ramig RF. The effects of host age, virus dose, and virus strain on heterologous rotavirus infection of suckling mice. Microb Pathog (1988) 4:189–202. 10.1016/0882-4010(88)90069-1 2848173

[21] Le Campion A Bourgeois C Lambolez F Martin B Leaument S Dautigny N Naive T cells proliferate strongly in neonatal mice in response to self-peptide/self-MHC complexes. Proc Natl Acad Sci U S A (2002) 99:4538–43. 10.1073/pnas.062621699 11917110 PMC123683

[22] Schonland SO Zimmer JK Lopez-Benitez CM Widmann T Ramin KD Goronzy JJ Homeostatic control of T-cell generation in neonates. Blood (2003) 102:1428–34. 10.1182/blood-2002-11-3591 12714521

[23] McNally A Thomson NR Reuter S Wren BW. ‘Add, stir and reduce’: *Yersinia* spp. as model bacteria for pathogen evolution. Nat Rev Microbiol (2016) 14:177–90. 10.1038/nrmicro.2015.29 26876035

[24] Bergman MA Loomis WP Mecsas J Starnbach MN Isberg RR. CD8(+) T cells restrict *Yersinia pseudotuberculosis* infection: bypass of anti-phagocytosis by targeting antigen-presenting cells. PLoS Pathog (2009) 5:e1000573. 10.1371/journal.ppat.1000573 19730693 PMC2731216

[25] Carey AJ Gracias DT Thayer JL Boesteanu AC Kumova OK Mueller YM Rapid evolution of the CD8^+^ TCR repertoire in neonatal mice. J Immunol (2016) 196:2602–13. 10.4049/jimmunol.1502126 26873987 PMC4779665

[26] You D Ripple M Balakrishna S Troxclair D Sandquist D Ding L Inchoate CD8^+^ T cell responses in neonatal mice permit influenza-induced persistent pulmonary dysfunction. J Immunol (2008) 181:3486–94. 10.4049/jimmunol.181.5.3486 18714021 PMC2659373

[27] Fernandez MA Evans IA Hassan EH Carbone FR Jones CA. Neonatal CD8^+^ T cells are slow to develop into lytic effectors after HSV infection in vivo. Eur J Immunol (2008) 38:102–13. 10.1002/eji.200636945 18081035

[28] Rudd BD Venturi V Smith NL Nzingha K Goldberg EL Li G Acute neonatal infections ‘lock-in’ a suboptimal CD8^+^ T cell repertoire with impaired recall responses. PLoS Pathog (2013) 9:e1003572. 10.1371/journal.ppat.1003572 24068921 PMC3771883

[29] Smith NL Wissink E Wang J Pinello JF Davenport MP Grimson A Rapid proliferation and differentiation impairs the development of memory CD8^+^ T cells in early life. J Immunol (2014) 193:177–84. 10.4049/jimmunol.1400553 24850719 PMC4065808

[30] Ruckwardt TJ Malloy AM Gostick E Price DA Dash P McClaren JL Neonatal CD8 T-cell hierarchy is distinct from adults and is influenced by intrinsic T cell properties in respiratory syncytial virus infected mice. PLoS Pathog (2011) 7:e1002377. 10.1371/journal.ppat.1002377 22144888 PMC3228797

[31] Venturi V Nzingha K Amos TG Charles WC Dekhtiarenko I Cicin-Sain L The neonatal CD8^+^ T cell repertoire rapidly diversifies during persistent viral infection. J Immunol (2016) 196:1604–16. 10.4049/jimmunol.1501867 26764033 PMC4744528

[32] Bantug GR Cekinovic D Bradford R Koontz T Jonjic S Britt WJ. CD8^+^ T lymphocytes control murine cytomegalovirus replication in the central nervous system of newborn animals. J Immunol (2008) 181:2111–23. 10.4049/jimmunol.181.3.2111 18641350 PMC4161464

[33] Belnoue E Fontannaz-Bozzotti P Grillet S Lambert PH Siegrist CA. Protracted course of lymphocytic choriomeningitis virus WE infection in early life: induction but limited expansion of CD8^+^ effector T cells and absence of memory CD8^+^ T cells. J Virol (2007) 81:7338–50. 10.1128/JVI.00062-07 17494081 PMC1933347

[34] Procario MC Levine RE McCarthy MK Kim E Zhu L Chang CH Susceptibility to acute mouse adenovirus type 1 respiratory infection and establishment of protective immunity in neonatal mice. J Virol (2012) 86:4194–203. 10.1128/JVI.06967-11 22345470 PMC3318603

[35] Sarzotti M Robbins DS Hoffman PM. Induction of protective CTL responses in newborn mice by a murine retrovirus. Science (1996) 271:1726–8. 10.1126/science.271.5256.1726 8596933

[36] Fadel SA Cowell LG Cao S Ozaki DA Kepler TB Steeber DA Neonate-primed CD8^+^ memory cells rival adult-primed memory cells in antigen-driven expansion and anti-viral protection. Int Immunol (2006) 18:249–57. 10.1093/intimm/dxh360 16418189

[37] Fadel SA Ozaki DA Sarzotti M. Enhanced type 1 immunity after secondary viral challenge in mice primed as neonates. J Immunol (2002) 169:3293–300. 10.4049/jimmunol.169.6.3293 12218149

[38] Ito S Ishii KJ Gursel M Shirotra H Ihata A Klinman DM. CpG oligodeoxynucleotides enhance neonatal resistance to *Listeria* infection. J Immunol (2005) 174:777–82. 10.4049/jimmunol.174.2.777 15634898

[39] Kollmann TR Reikie B Blimkie D Way SS Hajjar AM Arispe K Induction of protective immunity to *Listeria monocytogenes* in neonates. J Immunol (2007) 178:3695–701. 10.4049/jimmunol.178.6.3695 17339467 PMC2706399

[40] Chung H Pamp SJ Hill JA Surana NK Edelman SM Troy EB Gut immune maturation depends on colonization with a host-specific microbiota. Cell (2012) 149:1578–93. 10.1016/j.cell.2012.04.037 22726443 PMC3442780

[41] Surh CD Sprent J Homeostasis of naive and memory T cells. Immunity (2008) 29:848–62. 10.1016/j.immuni.2008.11.002 19100699

[42] Cho BK Rao VP Ge Q Eisen HN Chen J. Homeostasis-stimulated proliferation drives naive T cells to differentiate directly into memory T cells. J Exp Med (2000) 192:549–56. 10.1084/jem.192.4.549 10952724 PMC2193235

[43] McCarron M Reen DJ. Activated human neonatal CD8^+^ T cells are subject to immunomodulation by direct TLR2 or TLR5 stimulation. J Immunol (2009) 182:55–62. 10.4049/jimmunol.182.1.55 19109135

